# Programmable topological metasurface to modulate spatial and surface waves in real time

**DOI:** 10.1515/nanoph-2023-0490

**Published:** 2024-01-08

**Authors:** Qiang Xiao, Qian Ma, Yu Ming Ning, Long Chen, Shuo Liu, Jingjing Zhang, Jian Wei You, Tie Jun Cui

**Affiliations:** Institute of Electromagnetic Space, Southeast University, Nanjing 210096, China; State Key Laboratory of Millimeter Wave, Southeast University, Nanjing 210096, China

**Keywords:** topological metasurface, programmable metasurface, surface-wave modulation, spatial-wave modulation

## Abstract

We propose a programmable topological metasurface to integrate intelligent modulations of spatial and surface waves. A general design method is presented to design the programmable metasurface elements with PIN diodes. The surface waves can be controlled to propagate along the topological domain-wall interface by programming the C_3_-symmetry elements, while the spatial waves are modulated by the patterns of C_6_-symmetry elements. By independently controlling the bias voltages of meta-elements, the programmable topological metasurface can generate different coding patterns with distinct combinations of C_3_- and C_6_-symmetries in real time, respectively achieving the dynamical manipulation of the surface- and spatial-wave by using distinct element states in a time-division manner. To validate the modulation performance, we perform both near- and far-field tests with different coding patterns. Experimental results demonstrate good agreement with numerical simulations, thereby showcasing the flexible manipulations of surface waves and spatial waves by the topological metasurface. The proposed metasurface opens up new possibilities for multifunctional metadevices, which hold great potentials for future wireless communications and smart sensing systems.

## 1 Introduction

Metasurfaces are composed of periodic or quasi-periodic subwavelength artificially engineered structures, which have increasingly attracted great attention due to the excellent regulation of electromagnetic (EM) waves [1], [2], [3], [4]. The proposition of the generalized Snell’s law [5] has revolutionized the manners of EM manipulations by introducing abrupt phases on metasurfaces, hence leading to the development of a great number of novel EM devices and applications such as cloaks [6], [7], anomalous polarization converters [8], sensing [9], and perfect lens [10], [11]. In 2014, the concept of digital coding and programmable metasurfaces was proposed with great innovation to tailor the EM wavefronts more flexibly in programmable ways [12], which also bridges the EM physical world with the information world by digital processing methods [13], [14]. This concept is further promoted to information metasurfaces [15], [16], [17]. More importantly, the programmable metasurfaces are designed with many periodic meta-elements loaded with active devices like varactors and positive-intrinsic-negative (PIN) diodes to dynamically manipulate the EM wavefronts in real time. The coding sequences corresponding to different EM functions can be pre-computed and stored in a field programmable gate array (FPGA). Consequently, diverse EM phenomena, functions, devices, and systems can be fastly accomplished by the programmable method, such as space-time metasurfaces [18], [19], brain-computer metasurfaces [20], [21], [22], and nonreciprocal devices [23], [24], [25]. Furthermore, the exponential advancement of artificial intelligence (AI) technology has enriched the research on intelligent metasurfaces, leading to a wide range of scenarios such as intelligent imagers [26], [27], self-adaptively smart metasurfaces [28], [29], and physical diffractive deep neural networks (D^2^NNs) [30], [31], [32].

Recently, inspired by the discovery of topological states of matter in condensed matter physics and the development of topological phases [33], [34], researchers have been continuously exploring similar effects in classical and bosonic systems. This exploration has yielded fruitful results in photonics, particularly drawing significant attention to topological photonics [35], [36]. The roots of topological photonics can be traced back to the imitation of the quantum Hall effect, in which quantum Hall edge states could be emulated using light along an interface between two magneto-optical photonic crystals with different topological properties, offering the potential for realizing unidirectional waveguides [37], [38]. As theories related to topological photonics have been developed, researchers have explored and investigated various intriguing applications, such as reconfigurable topological switches [39], [40], topological lasers [41], and unidirectional waveguides [42]. Two distinct properties of topological photonics (unidirectional and backscatter-immune propagation for defects and sharp bends) offer a powerful means of controlling the behavior of light. Consequently, the research in topological photonics has not only advanced the related physical theories, but also paved a way for more complex optical devices and applications [43], [44], [45]. The advanced research on intelligent metasurfaces has led to further developments of the topological photonics. However, most of the previous topological metasurfaces are focused on the surface-wave manipulations, but neglect the capacity to control the spatial waves. Integrated topological controls of surface waves and spatial waves may open a new perspective on the electromagnetic interaction mechanism and multi-function designs.

Inspired by our previously published work [40], here we propose reprogrammable controls of surface waves and spatial waves using a topological metasurface that can dynamically switch the propagating paths or scattered fields under normal incidence of EM waves. A general design method is presented to carefully design the programmable metasurface elements with PIN diodes. The resonance characteristic is observed based on the band diagram [46] and the programmable topological elements can be further designed and optimized to realize the 1-bit phase performance for spatial waves manipulation. We propose the programmable topological elements including C_3_- and C_6_-symmetry states to simulate and measure their reflection coefficients. The reflection phase difference of C_6_-symmetry elements is more than 140°, while the reflection phase difference of C_3_-symmetry elements is almost zero, both maintaining high reflection amplitudes. By applying different elements at different times, we design different coding patterns to demonstrate the regulation performance of surface- and spatial-wave when the topological metasurface is respectively excited by different feeding sources. Measured results are in good agreements with numerical simulations, confirming that the topological metasurface can dynamically manipulate both surface waves and spatial waves.

## 2 Results

### 2.1 Principle

**Figure 1:**
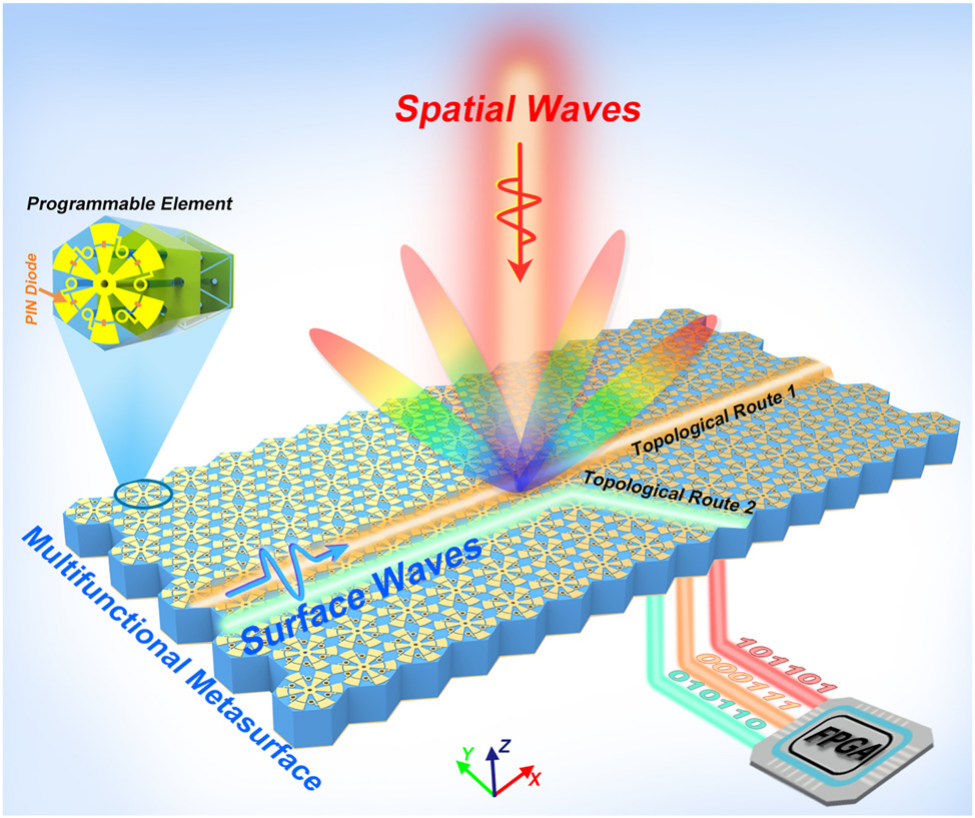
Schematical diagram of the multifunctional metasurface. By independently controlling the PIN diode states of each programmable element with FPGA, the multifunctional metasurface can dynamically switch the propagating topological routes for surface waves or generate different scattering beams for spatial waves. The proposed topological metasurface as a bridge organically integrates the intelligent modulation of spatial waves and surface waves.

As demonstrated in Figure 1, the schematic diagram of the proposed topological metasurface illustrates two programmable manipulation states: surface- and spatial-wave modulations. We present a general design method to design the programmable meta-elements integrating surface- and spatial-wave manipulation. The resonance characteristic can be observed in the band diagram while the surface-wave property is studied based on the energy band theory [46], balancing the performance of surface- and space-wave manipulation. The spatial wave manipulation can be realized by optimizing the resonance properties of the element. The proposed metasurface is composed of the above-designed meta-elements with different PIN diode states and the meta-elements are encoded into two categories: C_3_- and C_6_-symmetry states. As demonstrated in Figure S1, units 0 and 1 are programmed as C_6_-symmetry, while units 2 and 3 are C_3_-symmetry. In the surface-wave modulation mode, the proposed metasurface is precisely pumped by a continuous wave at the input port located on the metasurface. The surface waves are regulated to distinct propagation routes along the topological interfaces by constructing the topological domain-wall interfaces properly filled with C_3_-symmetry meta-elements, which are controlled by FPGA. For example, the straight and the 60-degree-bend propagation routes can be dynamically realized, as shown in Figure 1. To fully explore the EM properties of the proposed metasurface, spatial-wave manipulation is carefully studied based on the generalized Snell’s law. Firstly, the scattering performance of the metasurface can be artificially changed by switching different states of the C_6_-symmetry elements. In the spatial-wave modulation mode, the proposed metasurface is normally illuminated by a TE-polarized plane wave and then the incident spatial wave is scattered to free space for different beams based on the digital coding streams from FPGA. The spatial- and surface-wave modulation functions are together integrated into the proposed metasurface, offering a new perspective on compatible and reprogrammable wavefront manipulations. It should be noted that surface- and spatial-wave manipulation are executed separately in time domain by applying different unit states.

### 2.2 The general design method

**Figure 2:**
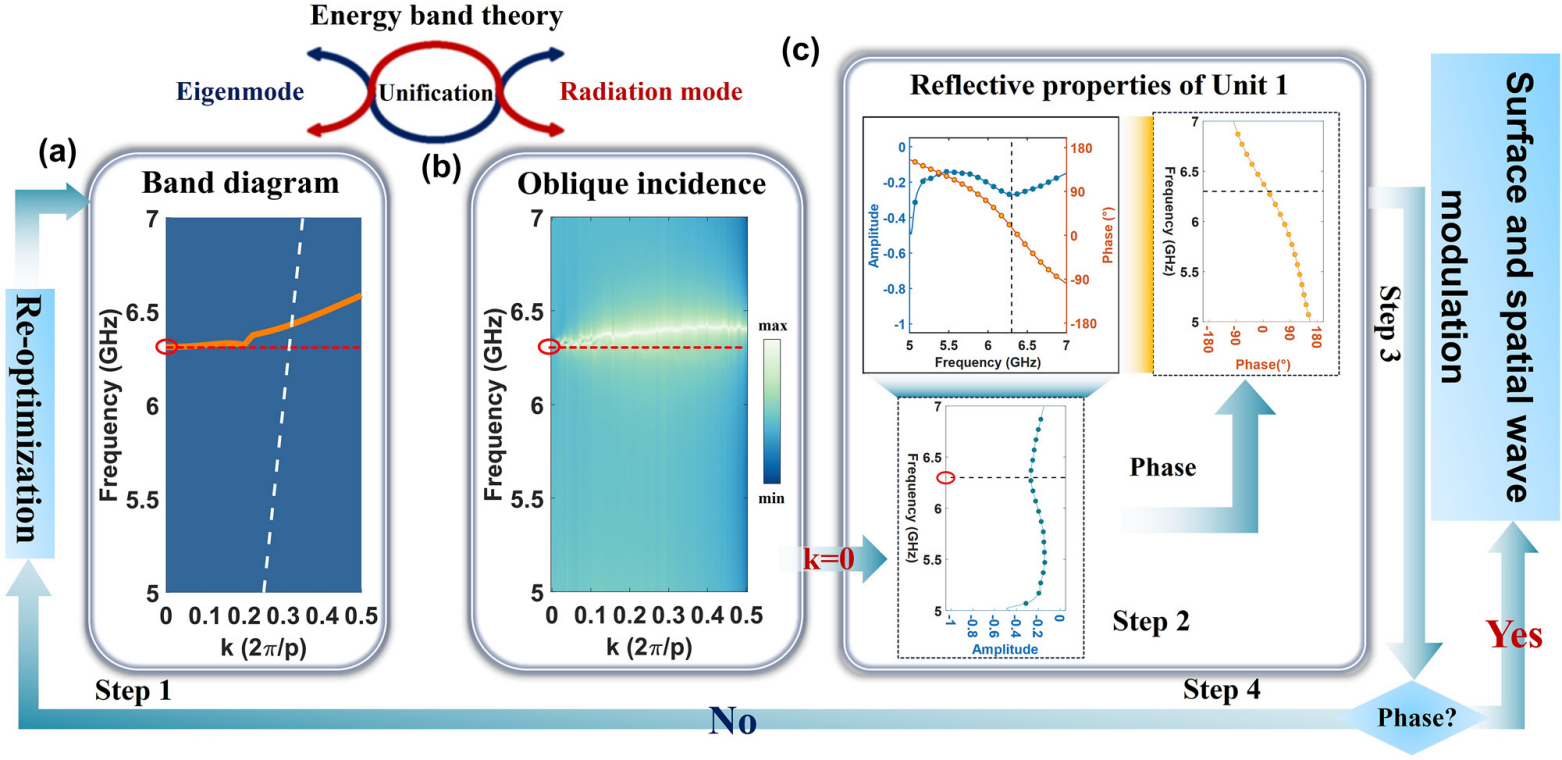
The flowchart of the proposed general design method. (a) The band diagram of unit 1. (b) The oblique incident performance of unit 1. (c) The reflective properties of unit 1 when a plane wave is normally incident.

To realize the integrated modulations of surface- and spatial-wave, a general design method is presented to design and optimize the programmable element based on the energy band theory, as shown in Figure 2. The band diagram is generally used to research surface wave characteristics and the spatial-wave manipulation is explored by the multimode resonance characteristic. The energy band theory, as a vital bridge, is considered to unify the band diagram for eigenmodes and the resonance characteristic for radiation mode during the design process of the element integrating surface- and spatial-wave regulation [46]. The detailed structure of the element is displayed in Figure S1. As an illustrative example, unit 1 is utilized to elaborately present the proposed method and the detailed design flowchart is clearly shown in Figure 2. The eigenmodes of unit 1 are firstly calculated by the commercial finite-element solver COMSOL Multiphysics and the simulated band diagram is shown in Figure 2a. The resonance characteristic is preliminarily observed because the point (wavevector *k* = 0) of the first Brillouin zone is highlighted and regarded as the normal incidence of a plane wave from the free space [46]. The oblique incident performance of the element has been further meticulously simulated in the commercial software CST Microwave Studio displayed in Figure 2b. By comparing Figure 2a and b, the resonance frequency occurs at around 6.3 GHz when the unit is normally illuminated by a plane wave from the free space. Hence, the resonance frequency can be roughly determined and optimized based on the band diagram, balancing the performance of surface- and space-wave regulation. Moreover, the 1-bit phase regulation performance is realized by adjusting the resonant frequency of elements and the process is re-optimized if the phase fails to meet goals, as displayed in Figure 2c. After careful optimization, the phase difference of digital coding states 0 and 1 is about 140° at around 6.2 GHz, proving the 1-bit phase control performance of the designed coding elements. The proposed method can be utilized to guide the fast design and optimization of topological metasurfaces with both surface and spatial waves manipulation, enabling multifunctional metasurface devices.

### 2.3 Simulated results

**Figure 3:**
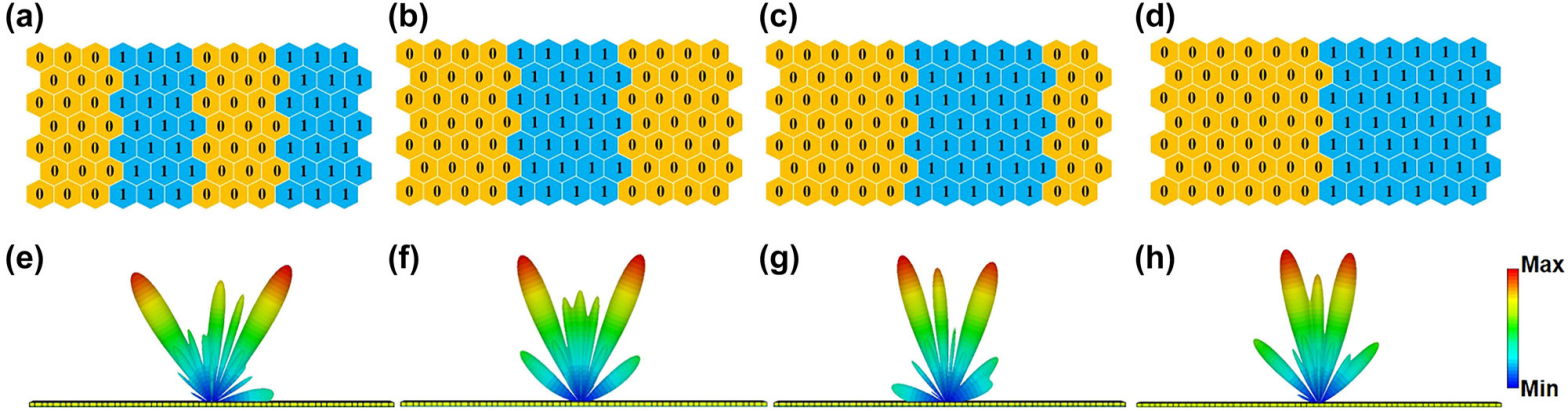
Simulated far-field results corresponding to the four periodic coding sequences. (a–d) Coding patterns of periodic coding sequences “000111…”, “00001111…”, “0000011111…”, and “000000111111…”, respectively. (e–h) Simulated 3D scattering patterns corresponding to the top coding patterns in (a–d).

The spatial waves can be manipulated to directional beams with different deflected angles when the metasurface is encoded with gradient phase distributions and is vertically illuminated by the TE-polarized plane wave. According to the far-field scattering field theory of coding metasurface with *M* × *N* elements, the far-field scattering pattern can be written as:

(1) $$\begin{array}{ll} E\left(\theta ,\varphi \right) & =\overset{M}{\underset{i=1}{\sum }}\overset{N}{\underset{j=1}{\sum }}A_{ij}e^{-j\varphi_{ij}}\\ & \;\times e^{-jkp\sin\theta \left[\left(i-1/2\right)\cos\varphi +\left(j-1/2\right)\sin\varphi \right]} \end{array}$$

where *k* is wave vector from free space; *A*_*ij*_ and *φ*_*ij*_ are *M* × *N* matrices, representing the reflective amplitude and phase responses of the *M* × *N* coding elements under the illumination of TE-polarized EM waves; *p* is the period of the unit cell; *θ* and *φ* denote the elevation and azimuth angles of the scattering pattern in the far-field domain. According to the generalized Snell’s laws, the far-field scattering patterns can be changed by applying the different periodic lengths of the coding sequences. To verify the performance of spatial wave regulation by the proposed metasurfaces, the metasurfaces encoded with different periodic coding sequences are impinged by a TE-polarized plane wave, hence generating dual beams with different splitting angles. Four periodic coding sequences of coding elements 0 and 1 are successively shown in Figure 3(a)–(d) and the metasurfaces with corresponding coding sequences are performed with full-wave simulations in CST Microwave Studio, respectively. The simulated boundary conditions are set as open add space along *x*, *y*, and *z* directions. A TE-polarized plane wave normally illuminates the metasurface horizontally placed on the *XOY* plane and the far-field monitor is set as 6.2 GHz to calculate the far-field simulated results. Four far-field scattering patterns with different deflected angles corresponding to predesigned coding sequences are demonstrated in Figure 3(e)–(h). Limited by 1-bit phase manipulation, dual beams with different angles are generated when a plane wave is used as the excited source. As predicted, the proposed topological metasurface can regulate spatial waves by programming the C_6_-symmetry elements with 1-bit phase regulations.

### 2.4 Experimental verification

#### 2.4.1 The topological metasurface prototype

**Figure 4:**
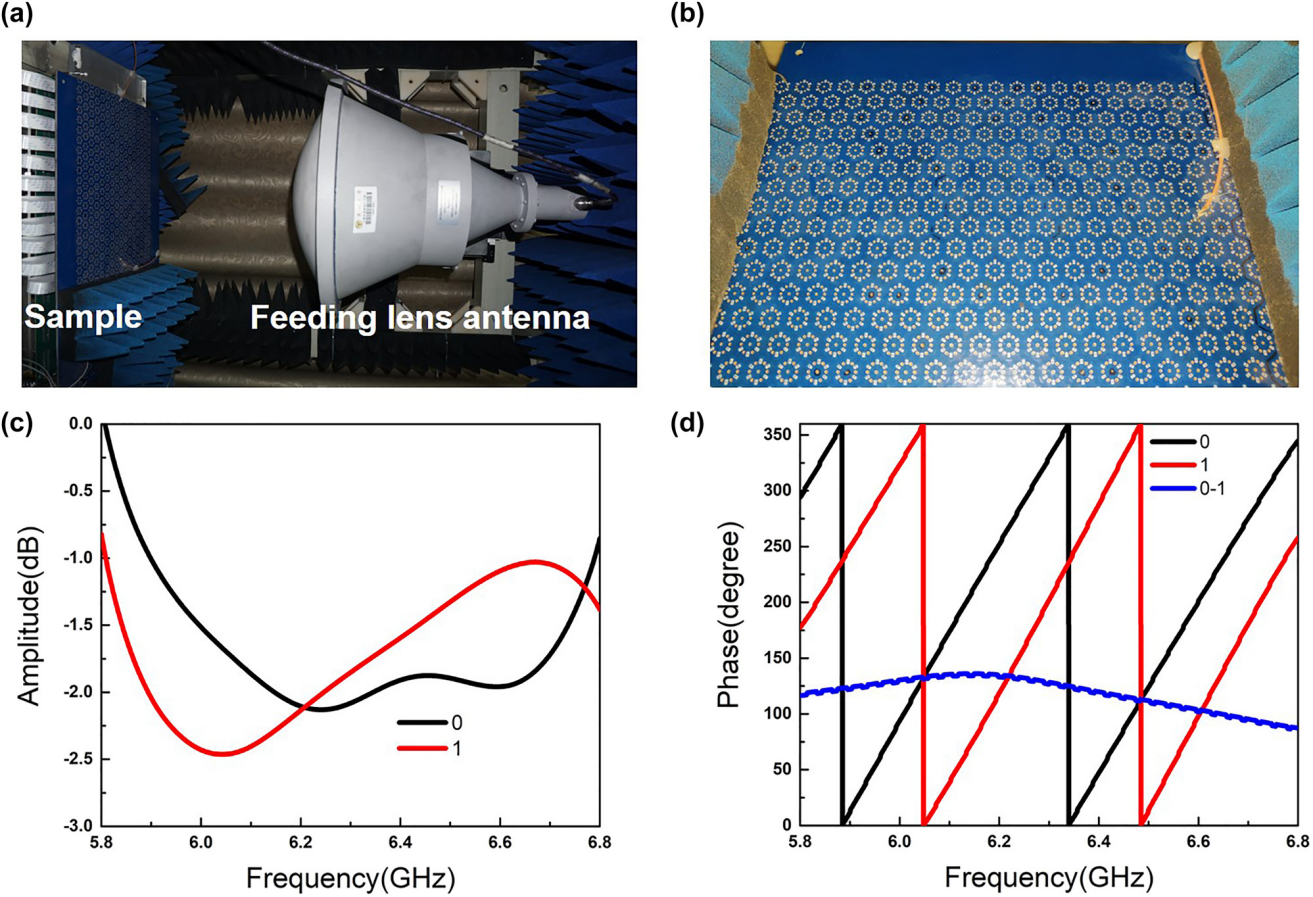
The experimental setup for reflection coefficients and photograph of the prototype. (a) The experimental setup is composed of a feeding lens antenna and sample. (b) The photograph of the prototype. (c, d) The measured reflective amplitude and phase responses of the coding element.

**Figure 5:**
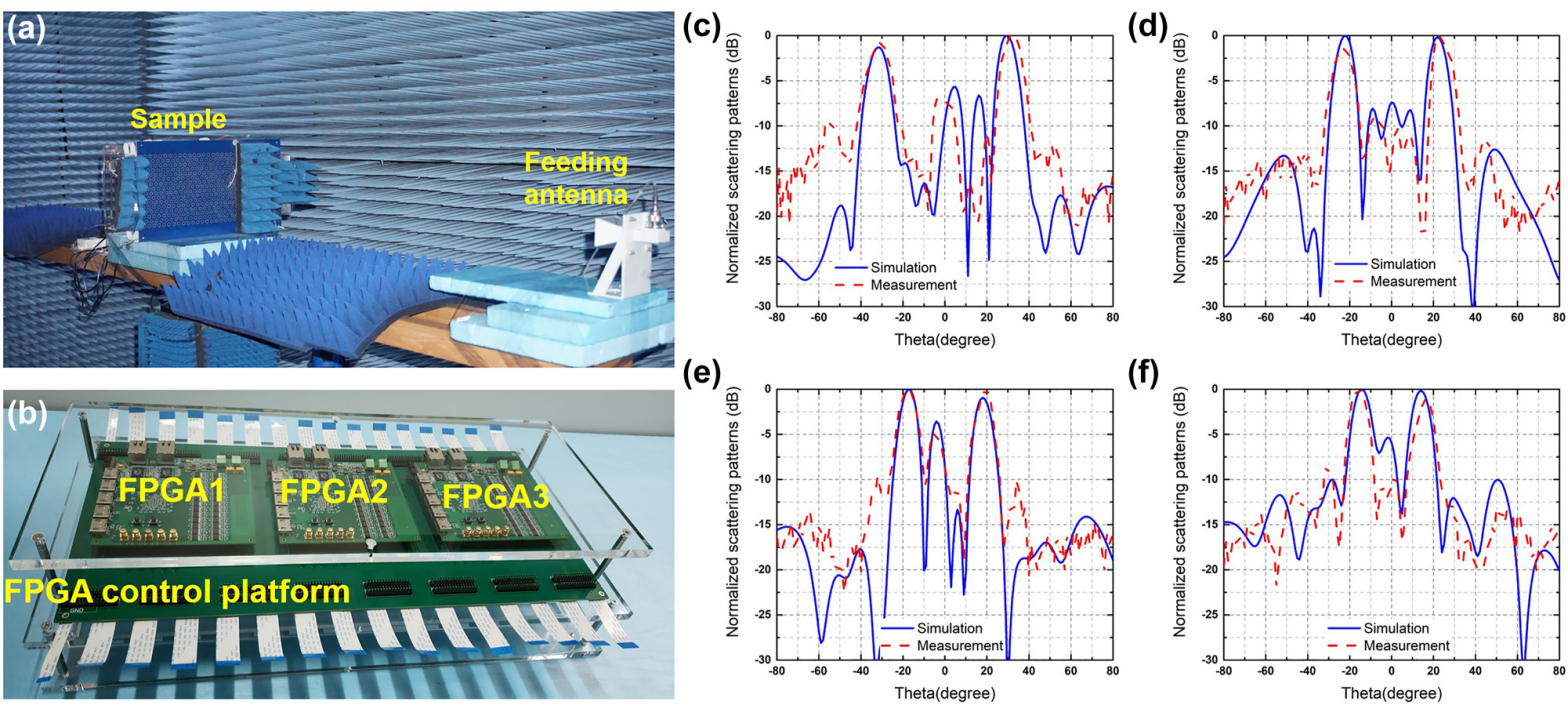
Measured far-field results for spatial wave manipulation. (a) The experimental setup in a microwave anechoic chamber. (b) The photography of the FPGA control platform. (c–f) The 2D far-field scattering results correspond to four coding patterns at 6.2 GHz.

To experimentally validate the integrated spatial- and surface-wave control performance of the proposed metasurface, we fabricate a prototype of the multifunctional metasurface using low-cost printed circuit board (PCB) technology to attain the illustrative examples. The prototype is composed of 488 coding meta-elements and has a total size of approximately 465 × 221 mm^2^. Firstly, the prototype is measured in a free space lens test platform, which consists of a feeding lens antenna, absorbent material, and a vector network analyzer (Agilent 5230C). As shown in Figure 4a, a feeding lens antenna is connected to one port of a vector network analyzer and generates plane waves to illuminate the metasurface sample. Hence, the reflective coefficient (S11) of the sample can be accurately acquired to evaluate the performance when all PIN diodes on the sample are simultaneously switched to ON or OFF states (C_6_-symmetry states) by multiple FPGAs. The photography of the sample embedded with PIN diodes is demonstrated in Figure 4b and the sample is electrically connected to a FPGA control platform with three FPGAs in Figure 5b. In fact, only 15 × 20 meta-elements are considered to regulate the spatial waves with the help of two FPGAs. The solder mask is applied to the surface of the metasurface, preventing the metal structures from being oxidized. The measured results with reflective amplitude and phase responses from 5.8 GHz to 6.8 GHz are illustrated in Figure 4(c) and (d) as the states of all meta-elements are changed from state 0 to state 1. Compared to the simulated scattering results of coding elements, the measured amplitude is higher than −2.5 dB and worse than the simulated. The deterioration of amplitude responses in the experiment may be caused by the error of the equivalent circuit model of the PIN diodes and measurement during the experiments. However, the reflective phase responses of C_6_-symmetry elements between the two states can keep a phase difference of about 140° at around 6.2 GHz, accomplishing the 1-bit phase-gradient modulations.

#### 2.4.2 Measured results for the spatial wave manipulation

The metasurface prototype is measured for the far-field scattering patterns in a standard microwave anechoic chamber, as shown in Figure 5a. The sample is accurately fixed on the FPGA control platform to form a convenient frame for easy placement on the turntable mount. A standard linearly-polarized horn antenna as a feeding source is vertically placed far away from the metasurface to generate TE-polarized quasi-plane waves. Due to the metasurface being placed along the TE-polarized direction, a standard broadband horn antenna with the TE-polarized direction is acted as a spatial energy receiver to receive the co-polarized far-field scattering signal on the other side of the microwave anechoic chamber. The distance between the receiving antenna and the sample is about 10 m to ensure far-field radiation conditions. Consequently, the far-field scattering results of the proposed metasurface can be measured at 6.2 GHz when the turntable mount loaded with the test sample is horizontally rotated 360°. All PIN diodes integrated on metasurface elements can be supplied with different voltages to be switched from ON to OFF state by an FPGA, hence dynamically realizing different coding sequences in Figure 3(a)–(d). The metasurface can be fastly reprogrammed by the FPGA control platform storing four different coding sequences of Figure 3(a)–(d) to modulate different scattering fields, respectively. The measured far-field scattering patterns are demonstrated in Figure 5(c)–(f) while the simulated results are plotted for comparison, respectively. It is obvious that the measured results have good agreement with the simulated results. However, some errors still exist in the experiments because of the following reasons: (1) the errors of prototype fabrication and soldering of PIN diodes; (2) the errors of manual configuration and measurement during the experiments; (3) the errors between RLC equivalent circuit models of PIN diodes and the experimental parameters; (4) the coupling effects between programmable elements. All measured results can effectively verify that the proposed reprogrammable topological metasurface can modulate the spatial waves according to the different coding sequences.

#### 2.4.3 Measured results for the surface wave manipulation

**Figure 6:**
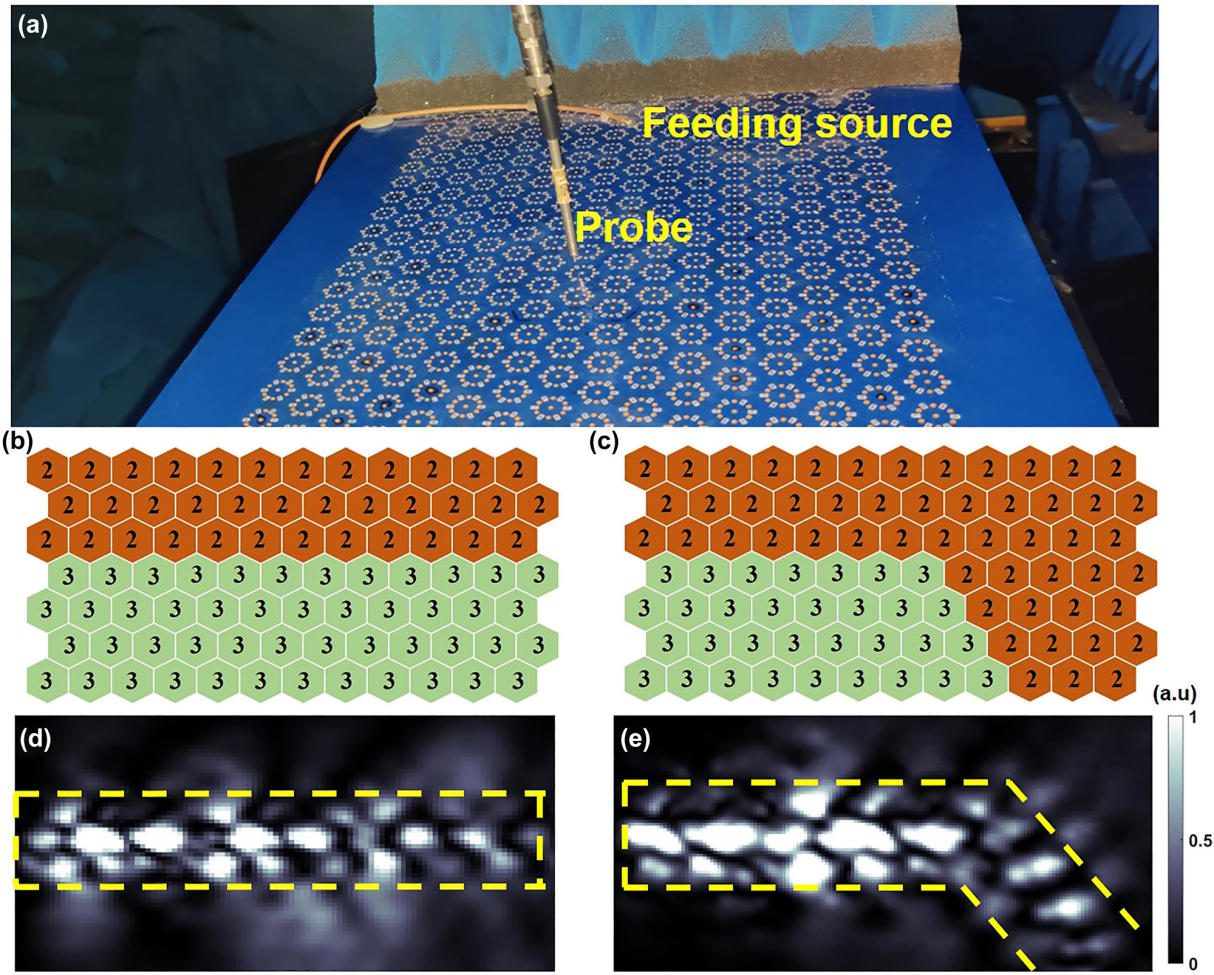
Measured near-field results for surface wave manipulation. (a) The near-field experimental setup in a microwave anechoic chamber. (b, c) The coding patterns are composed of coding elements 2 and 3 for topological straight and 60-degree-bend routes. (d, e) The measured near-field results of topological straight and 60-degree-bend routes at 7.2 GHz, respectively.

To demonstrate the flexible surface-wave regulations, the metasurface prototype is further measured for the near-field results with the test platform in Figure 6a. The coding patterns composed of C_3_-symmetry elements (units 2 and 3) for topological straight and 60-degree-bend routes are designed and shown in Figure 6(b) and (c). Based on the metasurface filled with coding meta-elements 2 and 3, the reprogrammable propagation routes are measured in the near-field microwave anechoic chamber and the test platform is displayed in Figure 6a. The near-field scanning probe connected with one port of a vector network analyzer is vertically placed 3 mm above the metasurface sample to automatically measure the intensity distribution. A coaxial line is welded on the sample and connected to another port of a vector network analyzer to excite surface waves. The measured results at 7.2 GHz in Figure 6(d) and (e) once again verify the excellent surface-wave control of the proposed topological metasurface as predicted. However, the energy loss exists in the surface waves propagation with a topological 60-degree-bend route because of the following reasons: (1) the energy loss is caused by a dielectric substrate F4B; (2) the active component PIN diodes are not ideal switching devices and the internal resistance can result in the energy loss; (3) the leakage of surface waves at the corner of the 60-degree-bend topological route. It can still be clearly observed that the surface waves can be controlled to propagate along the topological domain-wall interface (the straight and the 60-degree-bend topological routes) by programming C_3_-symmetry elements on the metasurface. Consequently, the proposed reprogrammable topological metasurface not only can dynamically switch topological propagation routes of surface waves but also can realize different scattering fields of spatial waves, accomplishing multifunctional EM waves modulation.

## 3 Conclusions

We propose a programmable topological metasurface and experimentally verify the intelligent modulation of spatial waves and surface waves in a time-division manner, enabling versatile EM manipulation. A general design method is presented to guide the fast design of topological metasurfaces with the integrated manipulation of surface and spatial waves. Firstly, the programmable topological elements including C_3_- and C_6_-symmetry states are simulated and measured for their reflective coefficients and the C_6_-symmetry meta-elements have been confirmed to have 1-bit phase regulation performance. As a proof of principle, four different coding patterns composed of C_6_-symmetry elements are designed to validate the spatial-wave manipulation performance of the topological metasurface and two distinct coding patterns composed of C_3_-symmetry elements for the surface-wave regulation. The measured results have shown good agreement with the numerically simulated ones, indicating the integrated manipulation of surface and spatial waves in a topological metasurface. The proposed topological metasurface as a bridge organically integrates the separate modulation of spatial waves and surface waves in the same metasurface platform, providing a new perspective on electromagnetic interaction mechanisms and multi-function designs. We believe that the reprogrammable topological metasurface has great potential in multifunctional EM metadevices, future wireless communications, and smart sensing systems.

## Supplementary Material

Supplementary Material Details
