# Supplementary material for: Programmable topological metasurface to modulate spatial and surface waves in real time
Source: Nanophotonics. 2024 Jan 8;13(12):2141–9. doi: 10.1515/nanoph-2023-0490 (PMC11501313; doi:10.1515/nanoph-2023-0490)
Supplement: Supplementary file 1 — Supplementary Material Details [file j_nanoph-2023-0490_suppl_001.docx]

Supporting Information

Programmable topological metasurface to modulate spatial and surface waves in real time

Qiang Xiao^†^, Qian Ma^†,^ *, Yu Ming Ning, Long Chen, Shuo Liu, Jingjing Zhang, Jian Wei You*, and Tie Jun Cui*

Supplementary Note 1: Design of the programmable elements

To realize intelligent modulations of spatial and surface waves, the entire structure of Figure S1(a) can be dynamically switched between the C_6_- and C_3_-symmetry. As displayed in Figure S1(a), the structure is composed of two substrate layers (S1, S2) and three metal layers (M1, M2, M3). The top metal layer (M1) composed of six Fan-shaped structures with six PIN diodes is placed in a hexagonal dielectric substrate F4B with a thickness of *h*1 = 3 mm (S1). The substate F4B has the relative dielectric constant of $\varepsilon_{r}$ = 2.65 and the dielectric dissipation angle tangent of tan *δ* = 0.001 (relatively low dielectric loss). The edge length of the hexagonal unit cell is *p* = 15$\sqrt{3}$ mm and the outer radius of Fan-shaped structures is *r1* = 7.5 mm with a middle slot of 0.3 mm, where six PIN diodes (MADP-000907-14020x from MACOM) are embedded. The equivalent circuit of PIN diodes is illustrated in Figure S1(e). The ON state of the PIN diode is equivalent to a series connection of a resistor with *R* = 2.2 Ω and an inductor with *L1* = 0.32 nH. The OFF state of the PIN diode is equivalent to a series connection of an inductor with *L2* = 0.4 nH and a capacitor with *C* = 40 fF. The middle part of the M1 layer is a metal circle with a radius of *r2* = 1.5 mm and connected to the metal ground (M2) by a metal via with a radius of *r3* = 0.6 mm. The outer arms of six Fan-shaped structures are applied with two different positive DC voltages from an FPGA because the odd (even) arms are connected to the bottom metal plate by six metal vias. The bottom metal plate (M3) is composed of a hexagon with an edge length of *l1 =* 2.08 mm and a trapezoid with a long edge length of *l2 =* 7.45 mm, as demonstrated in Figure S1(c). The radius of the holes in the central metal ground is 0.8 mm. A dielectric substrate (S2) FR4 ($\varepsilon_{r}$ = 4.3, tan *δ* = 0.025) with a height of *h2* = 0.2 mm is used as a prepreg layer to bond the M2 layer and the M3 layer. The M2 layer and the M3 layer are utilized to feed the PIN diodes of the M1 layer and the M2 layer also acts as the reflected ground to reflect the spatial waves. Finally, an FR4 layer with a thickness of 0.5 mm is set under the M3 layer to place the DC feeder lines.

The proposed elements with different diode states are programmed as four digital coding states 0, 1, 2, and 3, respectively, as shown in Figure S1d. To illustrate the scattering performance of the proposed unit cells under the illumination of the TE-polarized incident wave, full-wave simulations with unit-cell boundary conditions are performed in the commercial software Computer Simulation Technology (CST) Microwave Studio by the field-circuit co-simulation method. The simulated amplitude and phase responses of all coding elements from 5 GHz to 7 GHz are demonstrated in Figure S1(f, g). The amplitude responses of four coding elements are all greater than -1 dB from 5 GHz to 7 GHz, keeping higher reflective efficiency. The phase difference of digital coding states 0 and 1 is about 140$^{\circ}$ at around 6.2 GHz, proving the 1-bit phase control performance of the designed coding elements. The phases of the other two coding elements with *C*_6_-symmetry are almost the same and the meta-elements 0 and 1 have the *C*_6_-symmetry-protected Dirac points. Whereas the *C*_6_-symmetry of meta-elements 2 and 3 is degraded to *C*_3_-symmetry, and the corresponding Dirac points are gapped out to form bandgaps. As a result, meta-elements 2 and 3 with *C*_3_-symmetry are deliberately arranged to construct the programmable topological propagation interfaces of surface waves, and the other two coding meta-elements with *C*_6_-symmetry are used to modulate the spatial waves based on the generalized Snell’s law.


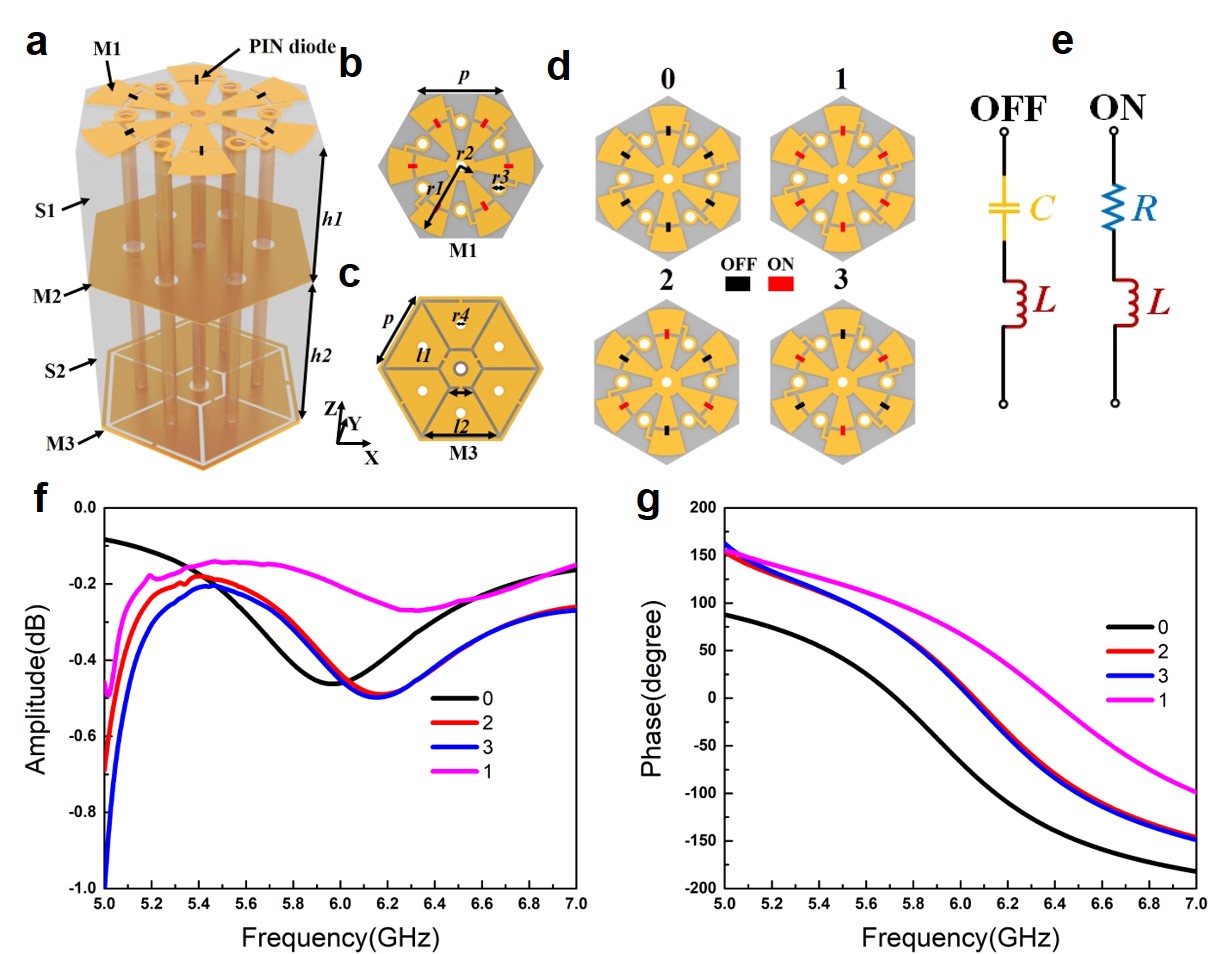


Figure S1. The reprogrammable coding element. (a) Sketch of the designed coding element. (b) The top view of the coding element. (c) The detailed structure of the bottom metal plate. (d) Four coding elements with different states of PIN diodes. (e) The equivalent circuit model of PIN diode with ON and OFF state. (f, g) The reflective amplitude and phase responses of the coding element under the incidence of a TE-polarized plane wave.
